# Supplementary material for: Chemical Diversity of Mediterranean Seagrasses Volatilome
Source: Metabolites. 2024 Dec 14;14(12):705. doi: 10.3390/metabo14120705 (PMC11677112; doi:10.3390/metabo14120705)
Supplement: Supplementary file 1 [file metabolites-14-00705-s001.zip › metabolites-3341634-supplementary.pdf]

Table S1 - Table of volatile organic compounds by biosynthetic pathway and relative abundance (± Standard deviation) in the four Mediterranean seagrass species extracted by HS-SPME and analyzed by GC-MS

Biosynthetic pathways	Compounds	CAS number	*C. nodosa*	*Z. noltei*	*P. oceanica*	*Z. marina*
Benzenoids	Benzaldehyde	100-52-7	0.01 ± 0.02	0.14 ± 0.07	0.03 ± 0.02	0.02 ± 0.01
	Benzeneacetaldehyde	122-78-1	0.11 ± 0.17	0.01 ± 0.04	0.14 ± 0.07	0.04 ± 0.09
	Benzyl alcohol	100-51-6	(-)	0.18 ± 0.40	0.01 ± 0.03	0.0(-).00
	Benzyl Benzoate	120-51-4	(-)	(-)	(-)	0.03 ± 0.04
	Diethyl Phthalate	84-66-2	(-)	0.14 ± 0.15	(-)	(-)
	Dihydroactinolide	15356-74-8	(-)	0.07 ± 0.15	0.13 ± 0.08	(-)
	Ethyl benzoate	093-89-0	(-)	(-)	0.06 ± 0.14	0.0(-).01
Fatty acid derivates	1-Heneicosene	1599-68-4	0.05 ± 0.09	(-)	(-)	(-)
	1-Heptadecene	6765-39-5	0.21 ± 0.40	0.31 ± 0.32	0.22 ± 0.22	0.15 ± 0.05
	1-Nonanol	143-08-8	(-)	0.03 ± 0.07	(-)	0.05 ± 0.06
	1-octanol	111-87-5	0.06 ± 0.15	(-)	0.17 ± 0.25	0.12 ± 0.18
	1-Pentadecene	13360-61-7	0.24 ± 0.42	0.13 ± 0.15	0.46 ± 0.12	0.13 ± 0.10
	1-tetradecene	1120-36-1	(-)	0.41 ± 0.31	(-)	(-)
	2(E)-octenal	2548-87-0	0.03 ± 0.06	0.06 ± 0.14	(-)	(-)
	2-Ethyl-3-hydroxyhexyl-2-methyl propanoate	74367-31-0	(-)	0.15 ± 0.21	0.03 ± 0.07	0.01 ± 0.03
	2-Ethylhexyl salicylate	118-60-5	(-)	(-)	0.09 ± 0.21	0.03 ± 0.07
	2-Methylbutyl laurate	93815-53-3	(-)	(-)	0.17 ± 0.30	0.11 ± 0.17
	2-penten-1-ol	1576-95-0	0.01 ± 0.02	(-)	0.25 ± 0.57	(-)
	3(E)-Hexen-1-ol	928-97-2	(-)	(-)	0.03 ± 0.05	0.03 ± 0.02
	3(Z)-Heptadecene	68155-0	0.55 ± 1.23	0.13 ± 0.31	0.12 ± 0.18	(-)
	3-ethyl-2-methyl-1,3-Hexadiene	61142-36-7	0.21 ± 0.26	0.23 ± 0.41	(-)	(-)
	3-Hydroxy-2,2,4-trimethylpentyl isobutyrate	77-68-9	0.07 ± 0.07	0.8(-).51	0.08 ± 0.12	0.06 ± 0.09
	6(Z),9(E)-Heptadecadiene	132833-58-0	0.09 ± 0.15	(-)	(-)	0.01 ± 0.02
	8-Heptadecene	2579-04-6	4.28 ± 4.20	0.11 ± 0.17	0.33 ± 0.09	1.62 ± 1.23
	9-Nonadecene	31035-07-1	0.06 ± 0.10	(-)	(-)	(-)
	Cyclotetradecane	295-17-0	0.05 ± 0.10	0.06 ± 0.09	(-)	(-)
	Decanal	112-31-2	0.28 ± 0.21	3.05 ± 1.57	1.45 ± 0.72	1.01 ± 0.15
	Dodecanal	112-54-9	0.19 ± 0.21	0.53 ± 0.29	0.37 ± 0.14	0.19 ± 0.05
	dodecyloxirane	3234-28-4	0.02 ± 0.03	(-)	0.03 ± 0.05	0.04 ± 0.03
	Eicosane	112-95-8	3.97 ± 2.53	0.02 ± 0.06	0.01 ± 0.03	0.04 ± 0.04

	Heneicosane	629-94-7	2.23 ± 1.58	0.15 ± 0.18	0.38 ± 0.19	0.55 ± 0.39
	heptadecane	629-78-7	12.8 ± 7.36	4.53 ± 3.28	46.0 ± 8.54	19.3 ± 4.37
	Hexadecanal	629-80-1	0.02 ± 0.05	0.05 ± 0.11	(-)	(-)
	hexadecane	544-76-3	0.55 ± 0.44	(-)	8.25 ± 0.67	0.88 ± 0.09
	Hexahydrofarnesyl acetone	68607-88-5	0.45 ± 0.56	0.52 ± 1.01	0.08 ± 0.11	0.07 ± 0.06
	Hexanal	66-25-1	0.01 ± 0.02	(-)	0.29 ± 0.27	(-)
	Isoamyl laurate	6309-51-9	0.03 ± 0.04	(-)	0.12 ± 0.28	0.53 ± 0.82
	Isopropyl myristate	110-27-0	25.6 ± 21.3	2.63 ± 2.07	2.42 ± 4.97	2.59 ± 5.02
	Isopropyl palmitate	142-91-6	0.04 ± 0.06	0.03 ± 0.07	0.04 ± 0.06	0.04 ± 0.04
	Methyl stearidonate	73097-00-4	(-)	(-)	0.28 ± 0.23	0.42 ± 0.25
	Nonadecane	629-92-5	12.2 ± 7.43	1.68 ± 0.79	4.70 ± 1.75	15.5 ± 7.20
	nonanal	124-19-6	0.03 ± 0.05	1.82 ± 2.40	0.93 ± 0.90	0.86 ± 0.63
	Nonanoic acid	112-05-0	(-)	(-)	0.35 ± 0.32	0.07 ± 0.15
	Octadecane	593-45-3	6.14 ± 6.44	0.57 ± 0.47	2.55 ± 0.69	0.97 ± 0.32
	Octyl caprylate	2306-88-9	0.56 ± 0.37	0.81 ± 0.32	2.92 ± 3.90	5.59 ± 8.27
	Octyl ether	629-82-3	0.32 ± 0.20	3.12 ± 1.92	1.1(-).29	1.13 ± 0.52
	Pentadecanal	2765-11-9	0.47 ± 0.46	7.55 ± 4.16	0.19 ± 0.27	0.04 ± 0.09
	pentadecane	629-62-9	2.94 ± 2.83	6.22 ± 2.63	8.76 ± 1.33	42.9 ± 7.14
	Tetradecanal	124-25-4	0.13 ± 0.13	1.11 ± 0.52	0.19 ± 0.05	0.1(-).02
	tetradecane	629-59-4	0.05 ± 0.08	0.89 ± 0.53	0.08 ± 0.05	0.18 ± 0.03
	Tetradecyloxirane	7320-37-8	(-)	(-)	0.29 ± 0.30	(-)
	Tridecanal	10486-19-8	1.07 ± 1.08	1.02 ± 0.46	0.34 ± 0.12	0.18 ± 0.20
Furanoid	2-Pentylfuran	3777-69-3	0.31 ± 0.44	0.5(-).82	(-)	(-)
Sulfur containing compound	DMS	75-18-3	16.9 ± 19.5	39.3 ± 25.7	3.52 ± 4.85	(-)
Terpenoids	2,6,10-Trimethyltridecane	3891-99-4	0.01 ± 0.03	(-)	0.03 ± 0.05	(-)
	2,6,11-trimethyl-Dodecane	31295-56-4	(-)	(-)	0.1(-).22	0.07 ± 0.16
	Alloaromadendrene	25246-27-9	(-)	0.73 ± 0.57	(-)	(-)
	Camphor	76-22-2	0.24 ± 0.18	(-)	(-)	(-)
	Citral	5392-40-5	1.23 ± 1.15	(-)	(-)	(-)
	Farnesan	3891-98-3	0.18 ± 0.10	(-)	(-)	(-)
	Farnesyl acetate	29548-30-9	0.14 ± 0.30	0.91 ± 0.70	0.74 ± 0.28	0.6(-).37

	Geranylacetone	3796-70-1	0.67 ± 0.57	3.42 ± 2.42	1.39 ± 0.69	1.13 ± 0.36
	Humulene	6753-98-6	0.1(-).05	(-)	(-)	(-)
	Isophorol	470-99-5	(-)	(-)	0.4(-).42	(-)
	Menthol	89-78-1	0.24 ± 0.33	(-)	0.07 ± 0.17	(-)
	Neral	106-26-3	0.86 ± 1.07	(-)	(-)	(-)
	trans-Caryophyllene	87-44-5	0.02 ± 0.02	(-)	(-)	(-)
	α-ionone	127-41-3	0.01 ± 0.04	3.36 ± 1.79	(-)	(-)
	β-cyclocitral	432-25-7	0.54 ± 0.38	2.36 ± 0.87	2.77 ± 1.51	0.43 ± 0.37
	β-Cyclohomocitral	472-66-2	(-)	0.06 ± 0.15	0.02 ± 0.04	(-)
	β-ionone	79-77-6	1.51 ± 1.06	6.75 ± 2.70	4.92 ± 2.15	1.51 ± 0.59
	β-Ionone epoxide	23267-57-4	0.07 ± 0.10	0.36 ± 0.57	0.58 ± 0.21	0.06 ± 0.09
	unknown_RT1193_i57	-	(-)	0.01 ± 0.03	0.02 ± 0.03	0.0(-).01
Unknown	Unknown_RT1452_i95	-	(-)	0.02 ± 0.06	0.05 ± 0.11	(-)
	Unknown_RT1625_i57	-	(-)	0.21 ± 0.25	(-)	(-)
	Unknown_RT1632_i55	-	(-)	(-)	0.07 ± 0.06	(-)
	Unknown_RT190_i57	-	(-)	(-)	0.36 ± 0.35	(-)
	Unknown_RT2498_i71	-	(-)	0.09 ± 0.12	(-)	(-)
	Unknown_RT2866_i149	-	(-)	0.07 ± 0.10	(-)	(-)
	Unknown_RT3024_i173	-	0.01 ± 0.04	0.07 ± 0.16	(-)	(-)
	Unknown_RT3129_i191	-	(-)	0.13 ± 0.18	(-)	(-)
	Unknown_RT3159_i197	-	(-)	0.05 ± 0.07	(-)	(-)
	Unknown_RT32_i57	-	0.02 ± 0.05	(-)	(-)	0.01 ± 0.03
	Unknown_RT3256_i55	-	(-)	(-)	0.03 ± 0.05	(-)
	Unknown_RT3330_i57	-	0.06 ± 0.09	0.06 ± 0.15	(-)	(-)
	Unknown_RT3355_i57	-	(-)	(-)	(-)	0.12 ± 0.12
	Unknown_RT3482_i227	-	0.22 ± 0.17	(-)	(-)	(-)
	Unknown_RT3605_i141	-	(-)	1.99 ± 0.92	(-)	(-)
	Unknown_RT3888_i127	-	(-)	(-)	0.04 ± 0.06	0.08 ± 0.15
	Unknown_RT4444_i79	-	(-)	(-)	0.07 ± 0.08	0.08 ± 0.12
	Unknown_RT600_i81	-	(-)	(-)	0.03 ± 0.05	0.0(-).00
	Unknown_RT715_i56	-	0.02 ± 0.05	(-)	0.03 ± 0.07	(-)

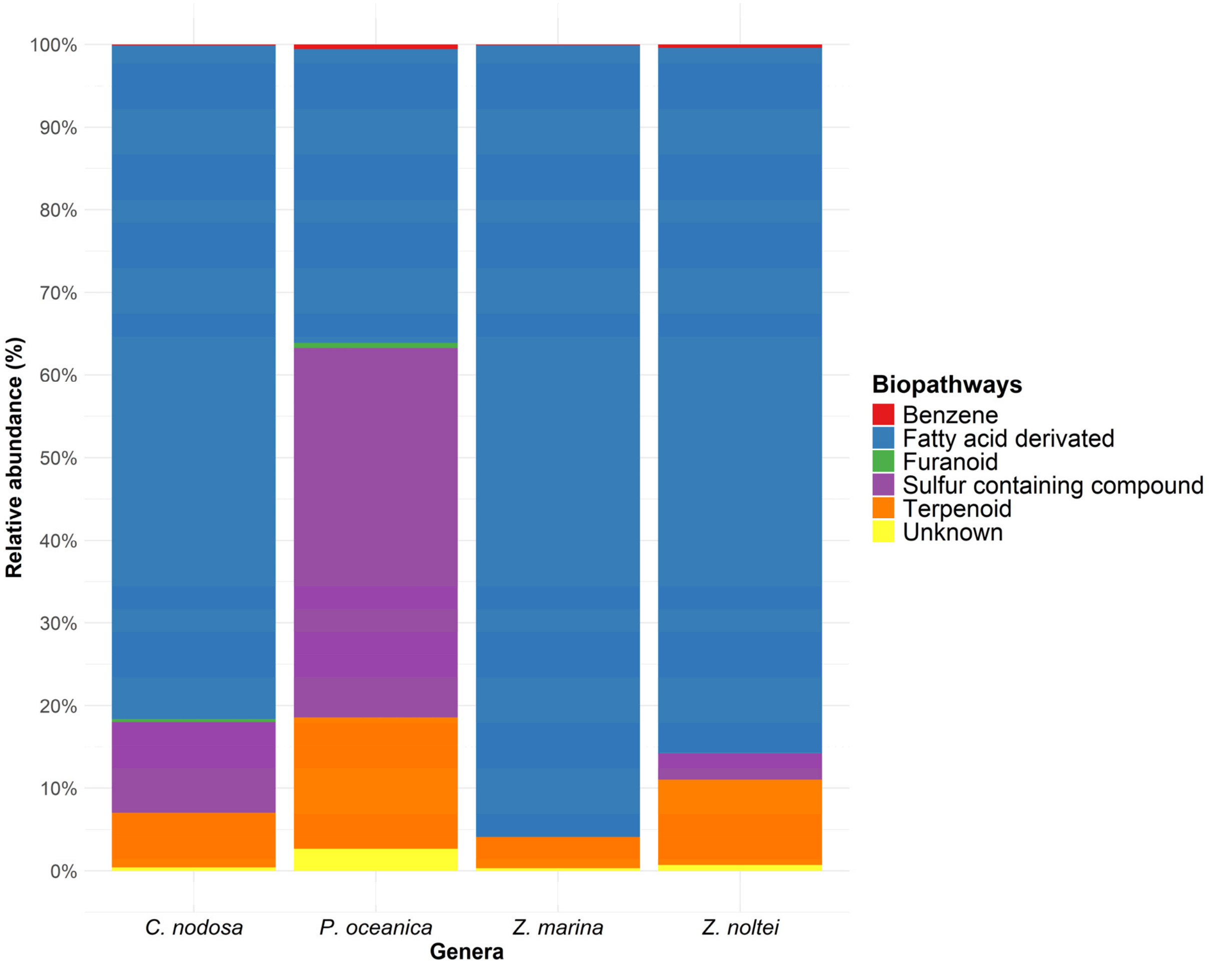

Figure S1 - Composition by biopathways of the volatilome in relative abundance of the four Mediterranean seagrasses

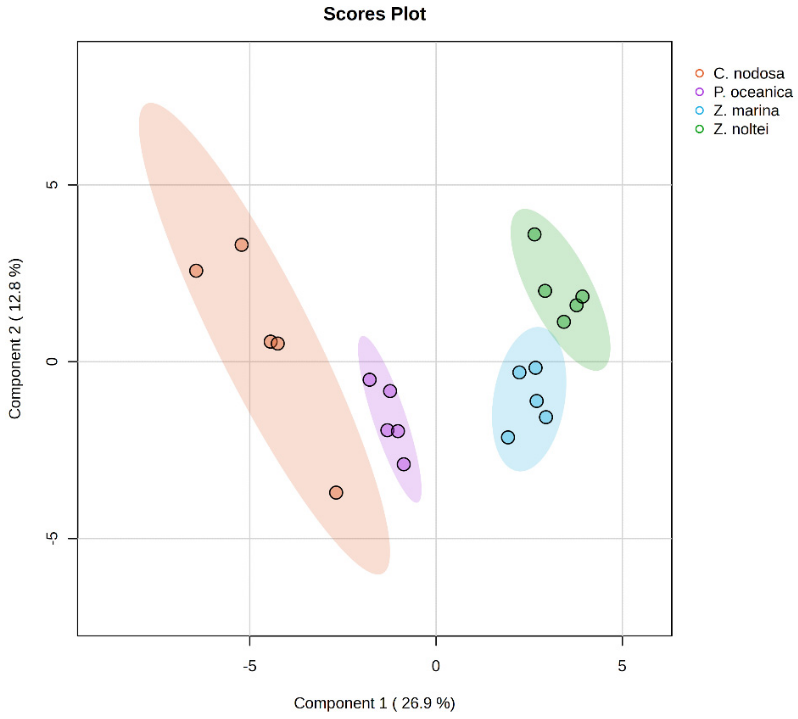

Figure S2 - PLS-DA of the volatile compounds of the four Mediterranean seagrass species

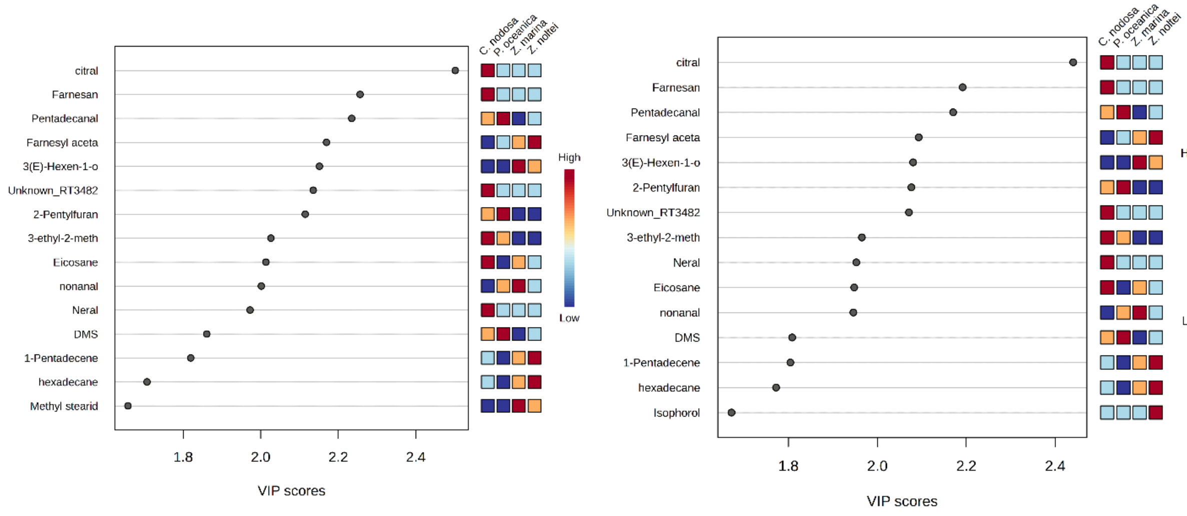

(a) (b)

Figure S3 - Most discriminant volatile organic compounds (Top 15) among the four Mediterranean seagrass species based on PLS-DA VIP scores of components 1 (a) and 2 (b).
